# Editorial for the Special Issue “Angiogenic Growth Factors in Tumor Development: Beyond New Blood Vessels Formation”

**DOI:** 10.3390/biomedicines12112541

**Published:** 2024-11-07

**Authors:** Camilla Palumbo, Loredana Albonici

**Affiliations:** Department of Clinical Sciences and Translational Medicine, University of Rome “Tor Vergata”, 00133 Rome, Italy; camilla.palumbo@uniroma2.it

A myriad of growth factors and receptors, whose effects are intertwined in complex interactions, do not only orchestrate tumor angiogenesis [1]. Tumor growth, progression, and therapy response are governed by the intricate interplay between angiogenic growth factors (AGFs), immune and stromal cells, and tumor cells [2,3]. It is becoming increasingly clear that this complex interaction network is challenging to disentangle. A thorough knowledge of the multiple cellular and molecular players involved is essential for rationalizing more effective anticancer strategies [4].

In this context, this Special Issue, titled “Angiogenic Growth Factors in Tumor Development: Beyond New Blood Vessels Formation”, includes two review articles providing an updated overview of current findings in the field. In particular, the review by Moshe and colleagues (Contribution 1) focuses on the role of AGFs in tumor immune escape. It discusses the growing body of evidence supporting the immunosuppressive effect exerted by different AGFs by acting on tumor-associated macrophages, myeloid-derived suppressor cells, dendritic cells, T cells, and T regulatory cells (Tregs). The authors also review the currently available antineoplastic agents in various stages of preclinical/clinical development targeting AGFs, their receptors, or key downstream effectors and discuss the therapeutic potential of combining anti-angiogenic agents with chemotherapy or immune checkpoint inhibitors (ICIs).

The potential of combining anti-angiogenic agents and immunotherapy is also discussed in the second review (Contribution 2), specifically in the context of clear cell renal cell carcinoma (ccRCC). The authors point out that ccRCC represents a paradigm for treatment with anti-angiogenic agents due to its highly angiogenic nature [5,6]. However, the efficacy of these agents is limited by a variety of intrinsic and acquired resistance mechanisms, which are comprehensively outlined in their paper. In this regard, particular emphasis is placed on the impact of intratumoral fibrosis on tumor growth and treatment resistance and the role played by VEGF and other pro-angiogenic factors, particularly TGFβ and CTGF, in promoting the accumulation of fibrotic material in the tumor microenvironment, ultimately favoring cancer aggressiveness and immune escape.

In addition to these review articles, this Special Issue comprises four research articles. The first of them (Contribution 3) retrospectively investigates the expression levels of angiogenic and immunological markers in tumor samples obtained from a cohort of patients with prostate cancer (PCa), including patients who did and who did not develop metastasis (PCM+ and PCM- group, respectively) during the 5-year follow-up period. The reported results first demonstrate that intratumoral PlGF expression is higher in the PCM+ than in the PCM- group, suggesting that the levels of this AGF in primary PCa may improve the accuracy of metastasis prediction. Further, a positive correlation is found between the intratumoral expression of PlGF and both VEGFR1 and HIF-1α and between PlGF and the number of lymphocytes positive for the immune checkpoint inhibitory receptor PD-1. This latter finding supports the involvement of PlGF in favoring immunosuppression in the microenvironment of PCa.

Defining whether the status of specific AGFs may have a predictive value for the prognosis of the tumor and the response to therapy is an active field of research [4,7,8]. This aspect is also addressed in the article by Schirizzi and colleagues (Contribution 4), where *vegfa* gene copy number and protein overexpression are investigated as potential biomarkers for predicting the response to anti-angiogenic therapy with the VEGFR2-targeting monoclonal antibody Ramucirumab and paclitaxel (PTX) in patients with metastatic gastric cancer (mGC). The results of this study highlight that *vegfa* gene amplification is not always associated with the overexpression of the encoded protein. Furthermore, it is similarly prevalent in patients with rapidly progressing disease as in those achieving disease control following the Ramucirumab plus PTX therapy. Conversely, from the authors’ observation that intratumoral VEGFA expression levels are significantly higher in the disease control group, it emerges that the overexpression of this AGF may represent a biomarker able to identify patients with mGC who will mostly benefit from anti-angiogenic therapy.

Several lines of evidence have highlighted how angiogenesis and epithelial–mesenchymal transition (EMT) can concur in promoting tumor aggressiveness and how the same AGFs can drive tumor epithelial cells toward the acquisition of a mesenchymal phenotype with increased capabilities for migration and invasion [9,10]. In the research article by Romanzi et al. (Contribution 5), this topic is explored by using sferoids of hepatocellular carcinoma (HCC) or cholangiocarcinoma (CCA) cell lines treated with ANG-2 and VEGF, alone or in combination. Through this experimental approach, the authors demonstrate the ability of both AGFs and their combination to stimulate, albeit with cell line-dependent responses, the migration and invasion of HCC and CCA cells and induce EMT phenotypic markers expression.

Besides experimental biology studies, the mathematical modeling of different tumor features, including angiogenesis and sensitivity to anticancer agents, represents a powerful tool to describe the complexity of tumor biology and progression, as well as an instrument that can be used to develop predictive models that may aid in the optimization of anticancer treatments [11,12,13,14]. In this regard, in the research article by Nath et al. (Contribution 6), a mathematical modeling approach is applied to the problem of the nonlinear tumor response to anti-angiogenic agents, with the aim to maximize the efficacy of the treatment by minimizing tumor volume using an optimal drug dose.

We believe that the articles and reviews in this Special Issue can contribute to the knowledge of AGFs and their multifaceted role in tumor growth, progression, and response to therapy.
